# A Rare Case of Atezolizumab-Induced Very Severe Aplastic Anemia

**DOI:** 10.7759/cureus.93543

**Published:** 2025-09-30

**Authors:** Vamshi Vadlapatla, Rachel D Truong, Jennifer E Tseng

**Affiliations:** 1 Hematology/Oncology, Northwestern Medicine McHenry Hospital, McHenry, USA; 2 Hematology/Oncology, Northwestern University Feinberg School of Medicine, Chicago, USA; 3 Hematology/Oncology, Orlando Regional Medical Center, Orlando, USA; 4 Internal Medicine, Orlando Regional Medical Center, Orlando, USA; 5 Internal Medicine, University of Central Florida College of Medicine, Orlando, USA; 6 Hematology and Medical Oncology, Orlando Regional Medical Center, Orlando, USA

**Keywords:** anemia, aplastic anemia, atezolizumab, bone marrow failure, immune-checkpoint inhibitor (ici), immunotherapy-related adverse events, immunotherapy side effects, neutropenia, pdl-1 inhibitor, thrombocytopenia

## Abstract

Aplastic anemia is a rare bone marrow failure condition with pancytopenia and transfusion dependence. This condition is potentially fatal if left untreated. It is confirmed by bone marrow aspiration and biopsy showing diminished or absent hematopoietic precursors, with the severity of bone marrow failure ranging from non-severe to very severe based on the bone marrow cellularity and blood counts. This dreadful medical condition can be inherited, immune-mediated, or iatrogenic, with the latter category occurring due to direct damage to the hematopoietic stem and progenitor cells (HSPCs) in the bone marrow. The PD-L1 inhibitor atezolizumab is an immunotherapeutic medication that can cause aplastic anemia, albeit exceedingly rare, with only eight reports to date in the medical literature. We present a case of iatrogenic aplastic anemia from administration of the immune checkpoint inhibitor atezolizumab. This case report provides insight into the evaluation and management of very severe aplastic anemia secondary to atezolizumab and is, to our knowledge, the first reported case of iatrogenic aplastic anemia secondary to atezolizumab in an elderly female with non-small cell lung cancer (NSCLC).

## Introduction

Aplastic anemia is a well-known, albeit rare, condition that causes bone marrow failure and can range from non-severe to very severe, accompanied by pancytopenia. This condition is potentially fatal if left untreated. It is confirmed by bone marrow aspiration and biopsy showing diminished or absent hematopoietic precursors [1]. It can be inherited, immune-mediated, or iatrogenic, with the latter occurring due to direct damage of the hematopoietic stem and progenitor cells (HSPCs) in the bone marrow [1]. External agents such as pathogenic organisms, including parvovirus B19, certain medications, chemotherapy, radiation therapy, chemical compounds, and heavy metal poisoning are known to cause this disease [1].

Treatment of this condition varies from close observation to serial blood product transfusions and immunosuppression, depending on the underlying etiology and severity [2]. The PD-L1 inhibitor atezolizumab is an immunotherapeutic medication that can cause aplastic anemia, albeit exceedingly rare, with only eight reports to date in the medical literature [3-5].

We present a case of iatrogenic aplastic anemia from administration of the immune checkpoint inhibitor (ICI) atezolizumab. This case is, to our knowledge, the first case of iatrogenic aplastic anemia secondary to atezolizumab to be reported in an elderly female with non-small cell lung cancer (NSCLC).

## Case presentation

A 70-year-old female presented to the emergency department with complaints of intermittent fevers up to 101.3°F for seven days. She presented with fatigue, decreased appetite, dyspnea, lower extremity edema, right upper quadrant abdominal pain, nausea, and altered mental status. She has Stage IB (pT2aN0M0) pleomorphic carcinoma of the right lung, generalized anxiety, depression, and hyperlipidemia. Medications included buspirone, clonazepam, desvenlafaxine, fluoxetine, olanzapine, rosuvastatin, and topiramate. For her NSCLC, she underwent definitive right upper lobectomy with mediastinal lymph node dissection. The surgical specimen was positive for PD-L1 tumor proportion score (TPS) of 75%, and next-generation sequencing (NGS) testing was negative for alterations in ALK, MET Exon 14, NTRK, NRG1, RET, and ROS1.

We also obtained ctDNA testing, and no ctDNA was detected in her blood. She then received four cycles of adjuvant carboplatin/paclitaxel. This was followed by adjuvant atezolizumab (day 0).

On day 0, her white blood cell count decreased, absolute neutrophil count (ANC) decreased, hemoglobin decreased, and platelets were within normal limits. On Day +12 after administration of the first dose of atezolizumab, she started to develop severe joint pain, fatigue, fevers with Tmax 100.2°F, and decreased appetite. Blood work at this time was significant for decreased ANC, anemia, and thrombocytopenia. These symptoms progressively worsened, which led to her hospitalization for neutropenic fever on day +14. Her laboratory evaluation is summarized in Table 1.

**Table 1:** Patient's initial laboratory evaluation starting from the day she received atezolizumab (Day 0) BUN: Blood urea nitrogen; ALT: Alanine aminotransferase; AST: Aspartate aminotransferase; ALP: Alkaline phosphatase; WBC: White blood cells; RBC: Red blood cells; Hgb: Hemoglobin; Hct: Hematocrit; MCV: Mean corpuscular volume; MCH: Mean corpuscular hemoglobin; MCHC: Mean corpuscular hemoglobin concentration; RDW: Red cell distribution width; MPV: Mean platelet volume; ANC: Absolute neutrophil count

Lab	Day 0	Day +12	Day +14	Reference Range
Sodium	141 mmol/L	135 mmol/L	135 mmol/L	136–145 mmol/L
Potassium	3.8 mmol/L	3 mmol/L	3.6 mmol/L	3.5–5.1 mmol/L
Chloride	111 mmol/L	106 mmol/L	108 mmol/L	98–107 mmol/L
Bicarbonate	24 mmol/L	20 mmol/L	17 mmol/L	21–31 mmol/L
Glucose	112 mg/dL	140 mg/dL	134 mg/dL	65–100 mg/dL
BUN	12 mg/dL	18 mg/dL	19 mg/dL	7–25 mg/dL
Creatinine	0.89 mg/dL	1.23 mg/dL	0.82 mg/dL	0.6–1.2 mg/dL
Calcium	8.7 mg/dL	8.2 mg/dL	7.8 mg/dL	8.6–10.3 mg/dL
Total Protein	6.4 g/dL	6	5.4 g/dL	6.4–8.9 g/dL
Albumin	3.8 g/dL	3.5	3.3 g/dL	3.5–5.7 g/dL
Total Bilirubin	0.3 mg/dL	0.9 mg/dL	0.8 mg/dL	0.3–1.0 mg/dL
ALT	15 U/L	248	278 U/L	7–52 U/L
AST	21 U/L	284	315 U/L	13–39 U/L
ALP	62 U/L	117	201 U/L	34–104 U/L
WBC	2.9 x 10^3^/µL	1.7 x 10^3^/µL	1.6 x 10^3^/µL	4.4–10.5 x 10^3^/µL
RBC	3.25 x 106/µL	3.25 x 106/µL	2.74 x 106/µL	3.75–5.0 x 106/µL
Hgb	9.9 g/dL	10.1 g/dL	8.5 g/dL	11.4–14.7 g/dL
Hct	30.9%	30.8%	26.3%	34.3–45.5%
MCV	95.1 fL	94.8 fL	96 fL	80.5–99.8 fL
MCH	30.5 pg	31.1 pg	31 pg	26.8–33.0 pg
MCHC	32 g/dL	32.8 g/dL	32.3 g/dL	31.0–35.4 g/dL
RDW	18%	16.5%	16.8%	11.7–14.7%
Platelets	184 x 10^3^/µL	56 x 10^3^/µL	51 x 10^3^/µL	139–361 x 10^3^/µL
MPV	9.9 fL	13.8 fL	13.8 fL	8.9–12.7 fL
Lactic Acid	-	-	1.1 mmol/L	0.5–2.2 mmol/L
Neutrophil %	53.1%	43.6%	22.7%	42–75%
Band %	-	10.9%	2.1%	0–11%
Lymphocyte %	34.4%	29.7%	70.1%	20–44%
Monocyte %	11.9%	8.9%	2.1%	2–8%
Variant Lymphocyte %	-	5.9%	3.1%	0–7%
ANC	1.6 x 10^3^/µL	-	0.4 x 10^3^/µL	1.5–7.5 x 10^3^/µL

The workup for neutropenic fever, including imaging and cultures, was unrevealing for an infectious etiology for the fevers. She did, however, have transaminitis (>5×ULN) on blood work and right upper quadrant abdominal pain on physical examination.

Due to concern for grade 3 (Common Terminology Criteria for Adverse Events (CTCAE) grading v. 4.0) atezolizumab-related hepatitis, it was decided to discontinue immunotherapy further, and she was also started on prednisone 40 mg daily for this, with filgrastim-sndz and cefepime for febrile neutropenia. Her liver enzymes improved after this, but her neutropenia failed to improve despite receiving filgrastim at a dose of 480 mcg for five days, at which point her ANC was 0. It was then decided that further evaluation was needed to elucidate the etiology of her profound neutropenia. After an exhaustive battery of blood work, which was inconclusive for an etiology of this, she underwent a bone marrow evaluation with aspiration and biopsy on hospital day six (day +19 from administration of atezolizumab).

The bone marrow evaluation was negative for carcinoma involvement, and it revealed a severely hypocellular bone marrow with only 10% cellularity and marked myeloid hypoplasia. Flow cytometry showed a 10-15% cellular marrow with myeloid hypoplasia. Megakaryocytes were adequate in number with normal morphology. The erythroid lineage was normal, with progressive and terminal maturation. No monoclonal B or T cell populations were seen. The plasma cell population was polytypic, and no excess blasts were observed on either morphology or flow cytometry analysis. Iron stores were present but decreased, and no ring sideroblasts were seen.

Despite receiving filgrastim for seven days, she had no response, and her bone marrow was lacking myeloid precursors, so filgrastim was stopped. Instead, due to concerns of ICI-induced very severe aplastic anemia, she was started on weekly rituximab 375 mg/m² plus prednisone 80 mg daily as well as intravenous immunoglobulin (IVIG) for two days at the recommendation of the hematology multidisciplinary tumor board, with a plan to add cyclosporine to the treatment regimen if she failed to improve. Her total WBC count started to improve on her automated differential blood count, which began on hospital day 14, six days after the first dose of Rituxan administration.

**Figure 1:**
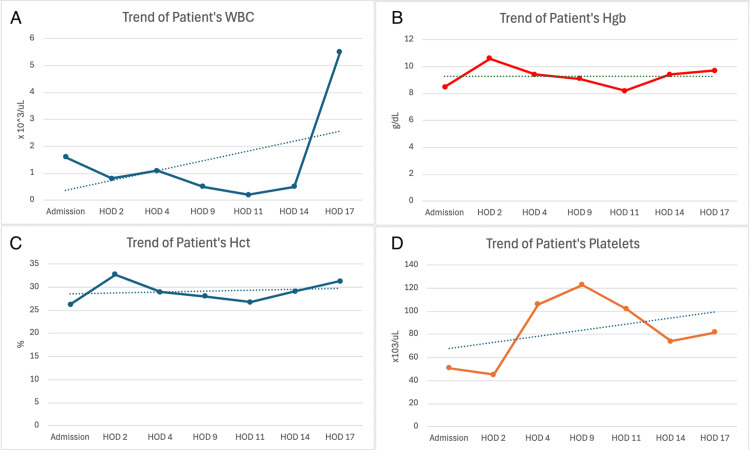
Trend of the patient's complete blood count over the course of her hospital stay Trends in the patient's (A) white blood cell count (WBC), (B) hemoglobin (Hgb), (C) hematocrit (Hct), and (D) platelet count. HOD: Hospital day

After two days of IVIG administration, her ANC was still 0. Filgrastim was restarted after the second weekly dose of rituximab on hospital day 17 (day +30 from atezolizumab administration). A day after reinitiation of G-CSF, her ANC increased to 700 cells/µL. This was continued for another day, after which her ANC normalized at 2700 cells/µL, and treatment was stopped. Throughout her hospital course, her infectious workup remained negative for an etiology for neutropenic fevers, her symptoms and blood work improved, and she was discharged the day after her ANC normalized. She continued to improve after discharge from the hospital and remained without a hospital readmission. Steroids were tapered in the outpatient setting while the patient was on PJP (*Pneumocystis jirovecii* pneumonia) prophylaxis. The patient's complete blood count (CBC) and differential throughout her stay are summarized in Figure 1 and Table 2.

**Table 2:** Summary of the patient's CBC and differential throughout her hospital stay HOD: Hospital day; WBC: White blood cells; RBC: Red blood cells; Hgb: Hemoglobin; Hct: Hematocrit; MCV: Mean corpuscular volume; ANC: Absolute neutrophil count; N/A: Not available

Lab	Admission	HOD 2	HOD 4	HOD 9	HOD 11	HOD 14	HOD 17	Reference Values
WBC	1.6	0.8	1.1	0.5	0.2	0.5	5.5	4.4–10.5 × 10^3^/µL
RBC	2.74	3.43	3.0	2.89	2.69	3.05	3.16	3.75–5.0 × 106/µL
Hgb	8.5	10.6	9.4	9.1	8.2	9.4	9.7	11.4–14.7 g/dL
Hct	26.3	32.8	29.0	28	26.8	29.1	31.3	34.3–45.5%
MCV	96.0	95.6	96.7	96.9	99.6	95.4	97.8	80.5–99.8 fL
Platelets	51	45	106	123	102	74	82	139–361 × 10^3^/µL
Lymphocyte %	70.1	91.0	91.2	93.9	N/A	79.2	8.0	20–44%
Monocyte %	2.1	1.0	3.6	5.5	N/A	17.6	8.0	2–8%
ANC	0.4	0.0	N/A	0	N/A	0	4.5	1.5–7.5 × 10^3^/µL
Notes	-	-	Bone marrow biopsy done	-	No diff due to WBC < 0.5	Zarxio restarted	Discharged	-

## Discussion

The United States Food and Drug Administration (FDA) has approved multiple ICIs for the treatment of solid tumors and hematologic malignancies [6]. Atezolizumab is one of these ICIs, carrying extensive warnings for adverse effects, including gastrointestinal distress, hepatic injury, nausea, vomiting, hair loss, rash, fever, dizziness, itchiness, and burning [7]. There have been reports of atezolizumab-induced single-cell line hematologic toxicity, hypophysitis, and cold agglutinin disease [4,8-13]. When choosing an immunotherapy regimen for NSCLC, a physician must therefore consider the risks of side effects versus the benefits of initiating treatment.

There have been several previous cases of single-cell line toxicity due to atezolizumab administration [8,9,11]. There was also a case of Evans' syndrome secondary to atezolizumab plus bevacizumab combination therapy [10]. In three of these cases, the patient's cell lines recovered after treatment with steroids or marrow-stimulating agents [9-11]. However, in one case, the patient did not recover despite aggressive treatment [8]. In addition, other ICIs, such as pembrolizumab, nivolumab, and durvalumab, have been shown to lead to aplastic anemia. However, our literature review found only one case report of iatrogenic aplastic anemia secondary to atezolizumab treatment, and seven cases were reported in the Adverse Event Reporting System (FAERS) when searching for "aplastic anemia" and "autoimmune aplastic anemia" [3-5]. Two cases listed in the FAERS were eliminated due to containing identical information in at least five categories (age, sex, reason for use, year reported, and regimen) and, therefore, being suspicious for repeated reporting. The demographics of these patients, along with the details of the cases, are listed in Table 3.

**Table 3:** Clinical characteristics of patients with iatrogenic aplastic anemia secondary to atezolizumab NSCLC: Non-small cell lung cancer

Source	Age	Sex	Year Reported	Reason for Use	Treatment	Outcome
FAERS Database [2]	Not specified	Not specified	2023	Malignant neoplasm	atezolizumab + nivolizumab + cemiplimab + avelumab + pembrolizumab + durvalumab + ipilimumab + tremelimumab	Died
FAERS Database [2]	66	Male	2021	NSCLC	atezolizumab + bevacizumab	Life-threatening
FAERS Database [2]	68	Male	2019	Neoplasm	atezolizumab	Disabled
FAERS Database [2]	67	Male	2019	NSCLC	atezolizumab	Life-threatening
FAERS Database [2]	Not specified	Male	2019	Unknown	atezolizumab + nivolumab	Other
Cheng et al. 2020 [3]	49	Male	2020	NSCLC	atezolizumab	Recovery
Guo et al. 2023 [4]	34	Female	2020	Stage IV cervical carcinoma	atezolizumab + bevacizumab + paclitaxel + carboplatin	Other
Guo et al. 2023 [4]	Not specified	Male	2017	Unknown	atezolizumab	Died

Of these cases, our patient is the only elderly female. She was also the only female being treated for NSCLC with atezolizumab. Our literature review found only one detailed case report of aplastic anemia secondary to atezolizumab, which occurred in a 49-year-old male with metastatic NSCLC [4]. His aplastic anemia presentation was after cycle six of atezolizumab, rather than after the first dose. The patient's bone marrow biopsy revealed less than 10% cellularity, with scattered lymphoid and erythroid cells, and no signs of dysplasia or missing trilineage marrow elements. Treatment focused primarily on immunosuppressive therapy with high-dose intravenous methylprednisone and anti-lymphocyte globulin for 10 days, a regimen similar to but more intense than that required by our patient. Notably, he did not receive filgrastim, and his aplastic anemia responded to his treatment 16 days after discovery. On the other hand, our patient's blood counts only started to improve on hospital day 14 after administration of rituximab and IVIG. It is entirely possible that the addition of filgrastim may have assisted our patient in her recovery, but the similarity in the length of time it took for her neutropenia to recover calls into question the efficacy of filgrastim in this case. It is also possible that this difference in treatment response could be due to other factors, such as the difference in severity of bone marrow hypocellularity, the later stage of the cancer, and differing treatment modalities.

Atezolizumab carries out its function by interfacing with programmed cell death-ligand 1 (PD-L1) [6]. This results in the blockage of inhibitory signals to CD8 T-cells and CD4 helper T-cells, thus suppressing the activation of the regulatory T-lymphocytes (CD4 and CD25) [6]. In addition, PD-L1 blockers also target inhibitory signals to B-cells, natural killer (NK) cells, and macrophages [6]. This results in the production of autoantibodies by B-cells, which can lead to anemia, thrombocytopenia, neutropenia, and bone marrow failure [6]. Direct bone marrow cytotoxicity can also result from the decrease of inhibitory signals to NK cells [6]. The above mechanisms clearly explain how our patient developed very severe aplastic anemia after beginning treatment with atezolizumab. This indicates that a physician should have a high index of suspicion for atezolizumab-induced aplastic anemia when faced with a patient undergoing treatment with immunotherapy presenting with new-onset hematologic abnormalities in multiple cell lines refractory to G-CSF administration.

Our case report has some limitations. Due to the single-case nature of this report, the generalizability of these findings is limited. In addition, we did not perform extensive autoimmune, genetic, or infectious testing to rule out all other potential causes of aplastic anemia. However, when considering the proximity of receiving atezolizumab to the patient's presentation, the previous reports of at least one cell line being decreased by atezolizumab administration, and the lack of an alternative explanation for the patient's condition, we strongly believe that atezolizumab is the primary cause of her aplastic anemia. Finally, there are drawbacks associated with the use of collecting data from FAERS, such as underreporting, false reports, incomplete reports, and inaccuracies. She was never rechallenged with ICI therapy.

This case report highlights the importance of weighing the risks and benefits when starting a regimen with an ICI, such as atezolizumab. While this medication is beneficial in patients with NSCLC, it has been shown in our case report to be a potential cause of aplastic anemia, a potentially lethal condition that could lead to complications such as bleeding, infections, transformation of the disease into lymphoproliferative disorders, or even death [14]. In addition, careful clinical and laboratory monitoring is required to ensure that bone marrow hematopoiesis failure due to checkpoint inhibitor therapy is recognized early and treated with immunosuppressive medications, such as steroids, rituximab, intravenous immunoglobulin, and bone marrow-stimulating agents. Our patient has made a full clinical and hematological recovery, and she continues to follow up with her oncologist. She has remained cancer-free with no evidence of disease for over 24 months so far.

## Conclusions

This case report highlights the importance of weighing the risks and benefits when starting a regimen with an ICI, such as atezolizumab. While this medication is well known to be beneficial in patients with NSCLC, it has been shown in our case report to be a cause of aplastic anemia, a potentially lethal condition that could lead to complications such as bleeding, infections, transformation of the disease into more severe myeloid disorders, or even death. In addition, careful clinical and laboratory monitoring is required to ensure that bone marrow hematopoietic failure due to checkpoint inhibitor therapy is recognized early and treated with immunosuppressive medications such as steroids, rituximab, intravenous immunoglobulin, and bone marrow-stimulating agents.

Our patient has made a full clinical and hematological recovery, and she also displays no evidence of active malignancy so far, as evidenced by serial clinical follow-up and negative Natera ctDNA testing. She continues to follow up with her oncologist and has remained cancer-free with no evidence of disease for more than 24 months so far.
